# Urban Water Storage Capacity Inferred From Observed Evapotranspiration Recession

**DOI:** 10.1029/2021GL096069

**Published:** 2022-02-08

**Authors:** H. J. Jongen, G. J. Steeneveld, J. Beringer, A. Christen, N. Chrysoulakis, K. Fortuniak, J. Hong, J. W. Hong, C. M. J. Jacobs, L. Järvi, F. Meier, W. Pawlak, M. Roth, N. E. Theeuwes, E. Velasco, R. Vogt, A. J. Teuling

**Affiliations:** ^1^ Hydrology and Quantitative Water Management Wageningen University Wageningen The Netherlands; ^2^ Meteorology and Air Quality Wageningen University Wageningen The Netherlands; ^3^ School of Agriculture and Environment University of Western Australia Crawley WA Australia; ^4^ Chair of Environmental Meteorology Faculty of Environment and Natural Resources University of Freiburg Freiburg Germany; ^5^ Foundation for Research and Technology Hellas Institute of Applied and Computational Mathematics The Remote Sensing Lab Heraklion Greece; ^6^ Department of Meteorology and Climatology Faculty of Geographical Sciences University of Łódź Łódź Poland; ^7^ Department of Atmospheric Sciences Yonsei University Seoul South Korea; ^8^ Korea Environment Institute Sejong South Korea; ^9^ Wageningen Environmental Research Wageningen University and Research Wageningen The Netherlands; ^10^ National Institute for Public Health and the Environment (RIVM) Bilthoven The Netherlands; ^11^ Institute for Atmospheric and Earth System Research / Physics University of Helsinki Helsinki Finland; ^12^ Helsinki Institute of Sustainability Science University of Helsinki Hesinki Finland; ^13^ Chair of Climatology Technische Universität Berlin Berlin Germany; ^14^ Department of Geography National University of Singapore Singapore Singapore; ^15^ Department of Meteorology University of Reading Reading UK; ^16^ Royal Netherlands Meteorological Institute (KNMI) De Bilt The Netherlands; ^17^ Independent Research Scientist Singapore Singapore; ^18^ Department of Environmental Sciences University of Basel Atmospheric Sciences Basel Switzerland

**Keywords:** urban climate, recession analysis

## Abstract

Water storage plays an important role in mitigating heat and flooding in urban areas. Assessment of the water storage capacity of cities remains challenging due to the inherent heterogeneity of the urban surface. Traditionally, effective storage has been estimated from runoff. Here, we present a novel approach to estimate effective water storage capacity from recession rates of observed evaporation during precipitation‐free periods. We test this approach for cities at neighborhood scale with eddy‐covariance based latent heat flux observations from 14 contrasting sites with different local climate zones, vegetation cover and characteristics, and climates. Based on analysis of 583 drydowns, we find storage capacities to vary between 1.3 and 28.4 mm, corresponding to *e*‐folding timescales of 1.8–20.1 days. This makes the urban storage capacity at least five times smaller than all the observed values for natural ecosystems, reflecting an evaporation regime characterized by extreme water limitation.

## Key Points

- A new method is applied to infer urban water storage capacity from evapotranspiration recession
- Our analysis of evaporation observations reveals water is limiting within days in cities worldwide
- Water storage capacity in cities is at least five times smaller than in natural systems

## 1 Introduction

With a large and growing share of the world population living in cities (United Nations, 2018), the impact weather‐related risks magnified by climate change, such as heatwaves and flooding (Wilby, 2007), also increases. In cities, air temperatures are typically higher than in the rural surroundings due to the Urban Heat Island effect (UHI; Oke, 1982; Oke et al., 2017; Santamouris, 2014). The UHI originates from the difference between the rural and urban energy balances due to lower albedo, radiation trapping, less vegetation, higher heat storage capacity and anthropogenic heat release (Oke, 1982). Because of its positive effect on evaporative cooling that is complemented by shading, urban vegetation is often given a central role in attempts to improve thermal comfort (Ennos, 2010). Indeed, higher vegetation fractions are associated with lower urban air and canopy temperatures (e.g., Gallo et al., 1993; Theeuwes et al., 2017; Weng et al., 2004), although in specific situations vegetation can cause higher temperatures (Meili et al., 2021). Wei and Shu (2020) showed that expanding the vegetation fraction as part of urban renewal can improve thermal comfort. However, vegetation‐mediated cooling strongly depends on water availability for evapotranspiration (ET; Avissar, 1992; Manoli et al., 2020).

The generally low ET over urban areas also reflects a different water balance that makes cities more prone to flooding. A high impervious surface fraction promotes storm water runoff, which can accumulate relatively fast (Arnold & Gibbons, 1996; Fletcher et al., 2013). Consequently, high runoff ratios decreases water availability for ET, and thus indirectly contributes to the UHI (Taha, 1997; Zhao et al., 2014). Heavy rainfall in cities can lead to flood volumes that are 2–9 times higher than in rural areas (Hamdi et al., 2011; Paul & Meyer, 2001; Zhou et al., 2019), often causing considerable damage (Tingsanchali, 2012). Solutions to problems related to the urban water and energy balance have been proposed under various names such as Water Sensitive Urban Design (Wong, 2006), Low Impact Development (Qin et al., 2013), Sustainable Drainage Systems (Zhou, 2014), Sponge Cities (Gaines, 2016), and Nature Based Solutions (Somarakis et al., 2019). All these concepts promote increasing infiltration and effective storage capacity, of which the latter is crucial for their performance (Graham et al., 2004; Qin et al., 2013). Therefore, methods to assess effective storage in cities at urban landscape scale are needed.

Estimation of the urban water storage capacity is challenged by the heterogeneity of sources for ET (Sailor, 2011). Previous studies have mainly focused on ET from individual sources (e.g., Gash et al., 2008; Pataki et al., 2011; Ramamurthy & Bou‐Zeid, 2014; Starke et al., 2010), as well as on their combined behaviour at street or neighborhood scale (e.g., Christen & Vogt, 2004; Jacobs et al., 2015; Meili et al., 2020, 2021). In order to study the ET on a neighborhood scale (order of hundreds of meters to 1–2 km), flux measurements through eddy covariance (EC) or scintillometry are becoming increasingly popular. Due to relatively large footprints, urban EC measurements often reflect a myriad of sources including impervious surfaces, vegetation, open water and all other sources of ET. Hence, in this paper an urban surface is defined as the entire urban landscape found within the footprint, rather than impervious surface only. This is in line with many studies on urban ET from an EC perspective, since the ET sources cannot be separated (e.g., Coutts et al., 2007b; Vulova et al., 2021). In contrast, modelling‐oriented studies are able to make this separation and thus often use urban and impervious interchangeably (e.g., Masson, 2000; Wouters et al., 2015). Examples of cities for which EC measurements have been studied are Arnhem (Jacobs et al., 2015), Basel (Christen & Vogt, 2004), Helsinki (Vesala et al., 2008), Melbourne (Coutts et al., 2007b), Seoul (Hong et al., 2019) and Singapore (Roth et al., 2017). Under water‐limited conditions, ET observations contain information on storage (Teuling et al., 2006). In one of the few studies directly linking urban ET and storage, Wouters et al. (2015) applied this principle to validate a new parametrization for the impervious contribution to urban water storage in Toulouse. However, the link between ET and footprint‐scale urban water storage remains largely unexplored.

Recession analysis can be used to link eddy‐covariance flux observations and storage properties. From the 1970s, discharge recession analysis has been extensively used in groundwater and hillslope hydrology (e.g., Brutsaert & Nieber, 1977; Kirchner, 2009; Troch et al., 2013). Similarly, daily ET values can be linked to water storage during a drydown, a period without precipitation creating water‐limited conditions. Assuming that the ET decay is exponential, the *e*‐folding time, or the timescale over which ET declines by 63%, reflects the available storage and resilience to droughts (Saleem & Salvucci, 2002; Salvucci, 2001; Wetzel & Chang, 1987). Since the storage is inferred directly from ET observations, this water storage is defined as the dynamic water storage capacity available to the atmosphere for ET, which includes soil moisture, intercepted precipitation, groundwater and open water varying from lakes to puddles. As a result of plant‐physiological processes, this storage is not necessarily constant (Dardanelli et al., 2004). In studies using daily ET over natural ecosystems, Teuling et al. (2006) and Boese et al. (2019) found timescales ranging from 15 days for short vegetation to 35 days for forest ecosystems, and corresponding storage capacities of 30–200 mm, with most sites in the range of 50–100 mm. A global‐scale analysis of surface soil moisture recession by McColl et al. (2017) found timescales ranging from 2 to 20 days. Although valuable insight can be obtained from a comparison of urban and rural ET dynamics, recession analysis has not yet been applied to urban ET.

This study extends the methodology developed by Teuling et al. (2006) to estimate footprint‐scale water storage capacity directly from EC observations of daily ET in cities without modeling ET itself. The methodology is applied to a new, unique collection of urban ET data containing cities in a range of climate conditions and with different urban land cover and structure. This allows for a first assessment of urban storage capacity across cities, an evaluation of how site characteristics (e.g., vegetation fraction) affect water storage, and a comparison of urban water storage to that of natural ecosystems.

## 2 Data and Methods

We analyze latent heat fluxes and auxiliary meteorological observations from eddy covariance flux towers at 14 sites in 12 cities to estimate water storage. Table 1 lists a number of important site characteristics, including key references. In these references, all observation sites and measurement details are fully described. The sites were selected based on the length of the data record (minimum of a year), flux footprints representing typical urban neighborhoods without other land covers, and the availability of observed precipitation and latent heat fluxes. All sites are located in reasonably flat terrain. Most sites were located in mid‐latitude climates, except Mexico City with a subtropical climate, Singapore with a tropical climate, and Helsinki, Łódź and Seoul with a continental climate. Vegetation fractions in the associated footprints vary between 6% and 56*%*.

**Table 1:** Site Characteristics and Summary of Regression Analysis

City	Lat. N (°)	Lon. E (°)	Köppen‐ Geiger climate	Avg. Temp. (°C)	Ann. Prec. (mm)	LCZ	*F*_*v*_ (%)	*z*_*s*_ (m)	*z*_*H*_ (m)	Start	End	Source	Drydown	Days	ET_0_ (mm d^−1^)	*λ* (day)	$t\frac{1}{2}$ (day)	*S*_0_ (mm)	*Mean R* ^2^
Amsterdam	52.37	4.89	Cfb	9.2	805	2	15	40	14	05–2018	10–2020	Ronda et al. (2017)	15	61	0.9–1.8 (1.4)	3.4–16.4 (4.5)	2.4–11.3 (3.1)	5.0–17.0 (7.3)	0.66
												Steeneveld et al. (2019)							
Arnhem	51.98	5.92	Cfb	9.4	778	2	12	23	11	05–2012	12–2016	Jacobs et al. (2015)	46	183	0.7–1.0 (0.8)	2.5–4.2 (3.0)	1.8–2.9 (2.1)	2.3–3.8 (3.0)	0.72
Basel (AESC)	47.55	7.6	Cfb	10	778	2	27	39	17	06–2009	12–2020	Lietzke et al. (2015)	120	500	0.8–1.0 (0.9)	4.2–5.6 (5.1)	2.9–4.0 (3.5)	3.6–4.9 (4.4)	0.75
Basel (KLIN)	47.56	7.58	Cfb	10	778	2	27	41	17	05–2004	12–2020	Schmutz et al. (2016)	158	661	1.0–1.2 (1.1)	4.9–6.8 (5.9)	3.4–4.7 (4.1)	5.4–7.8 (6.5)	0.72
Berlin (ROTH)	13.32	52.46	Cfb	9.1	570	6	56	40	17	06–2018	09–2020	Vulova et al. (2021)	7	33	0.4–0.9 (0.6)	4.8–11.0 (7.9)	3.3–7.6 (5.5)	1.3–9.9 (6.3)	0.67
Berlin (TUCC)	13.33	52.51	Cfb	9.1	570	5	31	56	20	07–2014	09–2020	Jin et al. (2020)	36	149	0.3–0.8 (0.5)	3.0–5.2 (3.7)	2.1–3.6 (2.6)	1.4–3.6 (3.0)	0.75
												Vulova et al. (2021)							
Helsinki	60.33	24.96	Dfb	5.1	650	6	54	31	20	01–2006	12–2018	Vesala et al. (2008)	45	202	1.2–1.8 (1.6)	3.7–6.1 (4.4)	2.5–4.2 (3.1)	6.0–11.0 (8.5)	0.78
												Karsisto et al. (2016)							
Heraklion (HECKOR)	35.34	25.13	Csa	17.8	464	3	12	27	11.3	Nov‐16	May‐21	Stagakis et al. (2019)	5	24	0.4–2.0 (0.5)	1.8–13.3 (6.5)	1.3–9.2 (4.5)	1.5–13.2 (2.8)	0.51
Łódź	51.76	19.45	Dfb	7.9	564	5	31	37	11	07–2006	09–2015	Fortuniak et al. (2013)	57	261	0.9–1.6 (1.3)	4.0–5.4 (4.4)	2.8–3.7 (3.1)	3.8–6.9 (5.8)	0.66
Melbourne (Preston)	−37.73	145.01	Cfb	14.8	666	5	38	40	6	08–2003	11–2004	Coutts et al. (2007b)	2	9	1.6–2.1 (1.9)	2.6–13.2 (7.9)	1.8–9.2 (5.5)	5.5–21.3 (13.4)	0.69
												Coutts et al. (2007a)							
Mexico City	19.4	−99.18	Cwb	15.9	625	2	6	37	9.7	06–2011	09–2012	Velasco et al. (2011)	8	49	0.7–1.5 (1.3)	5.5–16.5 (10.4)	3.8–11.5 (7.2)	5.8–21.9 (9.5)	0.65
												Velasco et al. (2014)							
Seoul	37.54	127.04	Dwa	11.9	1373	1	40	30	20	03–2015	02–2016	Hong et al. (2019)	10	59	0.6–2.0 (1.3)	2.3–9.9 (6.5)	1.6–6.9 (4.5)	3.3–10.7 (6.1)	0.56
												Hong et al. (2020)							
Singapore	1.31	103.91	Af	26.8	2378	3	15	24	10	03–2013	03–2014	Velasco et al. (2013)	7	40	1.3–1.6 (1.4)	4.6–20.1 (8.2)	3.2–14.0 (5.7)	7.7–28.4 (11.3)	0.81
												Roth et al. (2017)							
												Harshan et al. (2017)							
Vancoucer	49.23	−123.08	Csb	9.9	1283	6	35	28	5	05–2008	07–2017	Christen et al. (2011)	67	308	1.2–1.4 (1.3)	6.5–8.9 (7.3)	4.5–6.2 (5.1)	7.1–9.5 (8.3)	0.54

*Note*. The climate statistics are long‐term means (1999–2019). The indicated ranges for the parameters are the 5th and 95th percentile of the median distribution from the bootstrapping re‐samples with in brackets the median itself (LCZ Stewart and Oke (2012): 1 = compact high‐rise, 2 = compact mid‐rise, 3 = compact low‐rise, 5 = open mid‐rise, 6 = open low‐rise, *F*_*v*_: Surface fraction covered by vegetation in a 500 m radius around the measurement site, *z*_*s*_: Height of sensors above ground level, *z*_*H*_: Mean building height, ET_0_: Initial evapotranspiration, *λ*: *e*‐folding timescale, $t\frac{1}{2}$: Half‐life, *S*_0_: Effective, dynamic water storage capacity), *R* ^2^: Median goodness‐of‐fit.

Observations were reported in averaging periods of 10–30 min depending on the measurement protocol of each site. We used hourly averages to determine the timing of rainfall and 24‐hr averages for the recession analysis. For all sites the quality control of the observed heat fluxes was performed by individual researchers responsible for their ET flux observation site. Although the exact methodology of the quality control differs per site, all fluxes have been properly tested in accordance with procedures published in literature (Aubinet et al., 2012).

During multi‐day drydowns in urban areas without rainfall, runoff is typically minimal after a steep peak shortly after rainfall (Fletcher et al., 2013; Walsh et al., 2005). Therefore, the evolution in landscape‐scale dynamic storage (*S*) over the whole drydown can be simplified as:

(1) $$\frac{dS\left(t\right)}{dt}=-ET\left(t\right)$$

Under water‐limitation, daily ET becomes a function of storage. For impervious surfaces in cities, the storage dynamics have been described by a $\frac{2}{3}$‐power function resulting in depletion within a few hours of daytime (Masson, 2000; Ramamurthy & Bou‐Zeid, 2014). ET from other sources will likely show different behavior (Granger & Hedstrom, 2011; Nordbo et al., 2011), with ET from (urban) vegetation behaving more as a linear reservoir (Dardanelli et al., 2004; Peters et al., 2011; Williams & Albertson, 2004). Since impervious surfaces are typically quickly depleted, open water is constant and vegetation behaves more linear, we assume the flux footprint reflecting a mixture of different ET sources to effectively behave as a linear reservoir:

(2) ET(t)=f(S(t))=cS(t)

in which *c* = 1/*λ* is a proportionality constant. Combining Equations 1 and 2 and solving the differential equation leads to an exponential response of ET:

(3) $$ET\left(t\right)={ET}_{0}\exp\left(-\frac{t-t_{0}}{\lambda }\right)$$

where *λ* is the *e*‐folding timescale, and ET_0_ the initial ET. With these parameters the total dynamic storage volume *S*_0_ in mm that would be depleted during a complete dry down (*t* → *∞*) is given by:

(4) $$S_{0}=\int_{t_{0}}^{\infty }ET\left(t\right)dt=\lambda {ET}_{0}$$

so that *S*_0_ can be estimated by fitting observed ET in time during a drydown, without modeling the flux. Essentially, the storage capacity reflects the sum of water leaving the system as ET. Because of this direct inference without an imposed model structure, the shape of the fit has minimal influence on the results. To further tailor this concept to urban environments, the anthropogenic moisture flux can be included. This flux can contribute substantially to ET, in particular during long, dry periods (Grimmond & Oke, 1986; Miao & Chen, 2014; Moriwaki et al., 2008), and includes processes like transport, heating, cooling (indoor), human metabolism and irrigation, which do not directly depend on rainfall. Variation in the daily averages of these processes, except for irrigation, can be expected to be negligible over the course of one drydown. Thus, to account for these processes we added a constant base term to Equation 3. Since this yields parameters in compliance with the requirements explained below for only one drydown, we conclude that including this part of the anthropogenic moisture flux does not improve the physical representation of the city. As mentioned earlier, irrigation cannot be expected to be constant, while in some cities (e.g., Vancouver (Grimmond & Oke, 1986; Järvi et al., 2011) and Melbourne (Barker et al., 2011)) its contribution to ET can be considerable during long dry periods. We include two steps to prevent irrigation affecting the results. First we exclude irrigation by limiting drydowns to the first 10 days. This also reduces the influence of the smaller signal‐to‐noise ratio in the tail of the drydown on ET_0_. Second we require an *R* ^2^ > 0.3, in order to ensure a decreasing ET tendency reflecting storage as a main control on ET dynamics. The results converge until *R* ^2^ ≈ 0.3 (not shown), which shows drydowns with a lower *R* ^2^ are less reliable.

**Figure 1:**
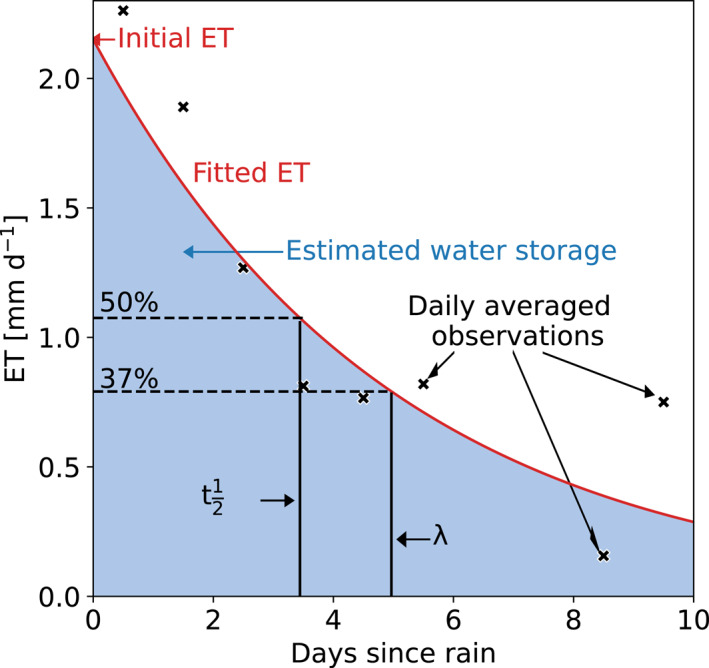
Illustration of the recession analysis. 24‐hour aggregated evapotranspiration versus the number of days following the last hour of precipitation for an example drydown from the Seoul data set with the fitted recession curve. Note that the fit was obtained by a linear fit on log‐transformed data (see Data and Methods). In the figure the parameters are indicated.

To estimate the parameters *λ* and ET_0_, we identified all periods without precipitation for at least three continuous days, the minimum requirement for an exponential fit (Figure 1). In order to preserve the information in ET during the first hours after rainfall (in case of low *λ*), we start the 24‐hr averaging bins directly after the rainfall event, regardless of its magnitude. The bin‐average is assigned to the middle of the day (e.g., the first bin is assigned to 0.5 days since rainfall). We exclude hours with an average shortwave incoming radiation below 10 W m^−2^ (i.e., nighttime), since nighttime ET tends to be low. No gap‐filling was applied, and only bins with at least 70% of data for daytime hours were analyzed. For the longest time series (Basel (KLIN)), requiring 70% instead of 100% increased the sample size by 48% respectively, while the median of the water storage capacities only changed by 25%. Further lowering the threshold did not increase data availability. Given the minimal effect on the results and potential to increase the sample size, 70% provides more information especially regarding cities with a shorter measurement period without compromising the results.

To allow for a variable timescale caused by a (seasonally) changing energy availability, we estimate *λ* and ET_0_ for every individual drydown. The parameter estimates result from linear fits (method of least squares) through the log‐transformed ET observations effectively applying Equation 3. In addition, the parameters are required to be physically plausible meaning positive *λ* and ET_0_, but below 35 days (maximum found by Teuling et al. (2006)) respectively 10 mm d^−1^. The maximum timescale prevents estimation of timescales much longer than the maximum drydown duration and storage estimates based on a limited dynamical range in ET. Given this filtering only excludes 10 cases, it does not influence our conclusions. Also, the average temperature during a drydown needs to exceed 0°C to exclude snow conditions, which is strict enough, confirmed by a check against snow records. To quantify the uncertainty of the estimated parameters, we applied bootstrapping using 5,000 re‐samples containing 90% of the estimates. The confidence interval is defined as the 5th and 95th percentile of the median distribution from the re‐samples.

With *λ* and ET_0_ the storage capacity is calculated according to Equation 4 (shaded area in Figure 1), as we assume the storage to be completely filled after every rainfall event. This assumption is supported by the absence of a dependency between the parameters and pre‐drydown rainfall. Drydowns from all seasons are included and analyzed for a seasonal effect, since the water storage available to the atmosphere may change due to for example, leaf phenology. Since it is not feasible to measure the water storage capacity in a complete urban footprint, this methodology offers the most direct estimation of the urban water storage. To investigate the possible impact of day‐to‐day variation or change in energy availability on the results, we repeated the recession analysis based on evaporative fraction (Gentine et al., 2007) multiplied by the average available energy over the drydown, which we included in the Supporting Information S1 (Table S1; Figures S1 and S2).

## 3 Results

**Figure 2:**
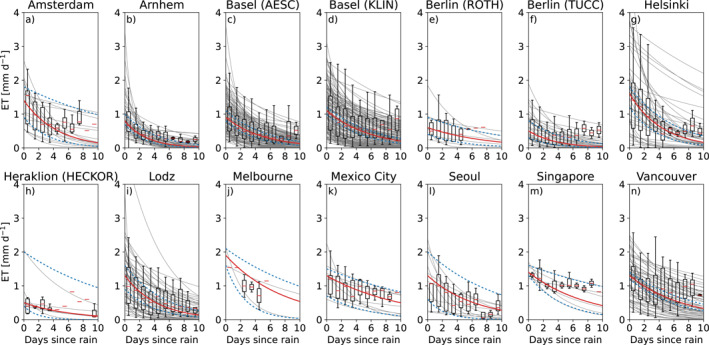
Daily average evapotranspiration versus the day since the last precipitation with in red (continuous) the recession curve using the median parameter values, in blue (dotted) the 5th and 95th percentile of the median distribution from the bootstrapping re‐samples, and in gray all individual drydowns. The boxplots show the spread of the observations. The parameters of the fitted curves are shown in Table 1. Since the parameters are based on individual drydowns, they do not necessarily follow the trend of the distributions.

In Figure 2, the individual drydowns (in gray) show a good resemblance of the characteristic behavior of the recession confirming the exponential behavior. In general, ET is quickly decaying within days after rainfall in all LCZ's represented in our sample, indicating urban ET is generally strongly limited by water availability even on the first day after rainfall. As all cities respond approximately similarly, this confirms the qualitative, decaying relation during a drydown. At some sites (e.g., Amsterdam), ET sometimes rises after 6–7 days, which is most likely due to higher ET rates during the fewer events of a duration longer than 6–7 days. The spread of the observations is higher than the uncertainty, which is the result of a seasonal dependency. The uncertainty is visibly higher in cities with shorter measurement periods, since shorter periods inevitably mean smaller samples of drydowns. For Arnhem, Basel (both), Berlin (both), Helsinki, Łódź and Vancouver, observations are available for more than two full years resulting in narrow uncertainty bands. Conversely, the uncertainty bands for the sites with records shorter than 2 years (Amsterdam, Melbourne, Mexico City, Seoul and Singapore) are as wide as the range of observations. In some panels (e.g., Amsterdam and Helsinki), we observe two groups of curves with distinct slopes, for which we found no explanation in seasonality, energy availability, temperature and pre‐drydown rainfall (amount and timing).

In Table 1, an overview of the parameters is given for the 583 drydowns that complied with all criteria. Of the total number of 1606 drydowns, 102 are excluded because of potential snow conditions. All drydowns had a positive ET_0_, and only three exceeded 10 mm d^−1^. 671 additional drydowns did not meet the minimum *R* ^2^ of 0.3. Finally, a negative *λ* led to excluding 237 drydowns and *λ* above 35 days to 10 more. The remaining drydowns have an *R* ^2^ of 0.69 and yielded initial evapotranspiration between 0.3 and 2.1 mm d^−1^ and *e*‐folding timescales between 1.8 and 20.1 days with the majority below 10.4 days, corresponding to half‐lives of 1.3–14.0 and 7.2 days. The related storage capacities appear to be between 1.3 and 28.4 mm with the majority below 13.4 mm. As mentioned before, the length of the measurement period determines the magnitude of the uncertainty, which for *S*_0_ varies from 1.2 mm in Basel (AESC) to 20.7 mm in Singapore.

**Figure 3:**
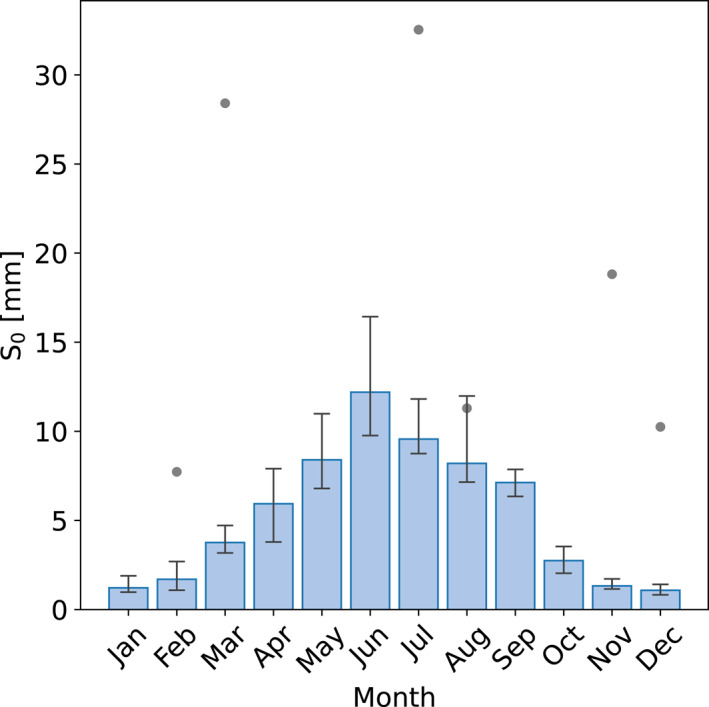
The seasonal dependency of the median *S*_0_ for the sites on the northern hemisphere (Melbourne is included shifted by half a year) in blue and for Singapore as gray dots. The uncertainty is determined similarly as in Figure 2.

For all sites, we find a considerable spread in the ET observations (Figure 2), which recurs in the estimated *S*_0_ values. In Figure 3, *S*_0_ is plotted against the month of the drydown, showing a very distinct seasonal dependency explaining why the spread in observations exceeds the uncertainty. Both ET_0_ and *λ*, on which *S*_0_ is based, show similar behaviour (not shown). Melbourne is shifted to fit the seasonality, as it is situated on the southern hemisphere. We expect that the enhanced effective storage capacity in summer is caused by increased vegetation activity. Since Singapore is close to the equator and its vegetation is evergreen, it is not expected to show seasonal effect, which is confirmed in Figure 3. Any connection between *S*_0_ and the site characteristics in Table 1 and climatic variables among which precipitation regime is overshadowed by the seasonal dependency covering the full range of *S*_0_ (Table 1), as we illustrate in Figures S3 and S4 in Supporting Information S1. It is unfortunately not possible to eliminate the influence of this dependency by focusing on one season due to the steep slope, and not by focusing on 1 month due to the low data density. Only after omitting half of the cities based on the number of drydowns, a relation between *S*_0_ and site characteristics is visible (Figure S5 in Supporting Information S1).

## 4 Discussion

In contrast to the results presented here for urban areas, Teuling et al. (2006) found timescales ranging from 15 to 35 days and storage varying between 30 and 150 mm for forests and grassland following a similar methodology. When compared to the urban parameter values (1.8–20.1 days and 1.3–28.4 mm), it is clear that both the timescales and storage capacities are much higher in rural areas. McColl et al. (2017) have analyzed soil moisture drydowns in a global study using satellite data with a resolution too coarse to explicitly resolve individual cities, thus resembling rural values. Although their timescales with values from 2 to 20 days are closer to ours, it must be noted the temporal resolution is one in every three days and their observations only regard the first few centimeters instead of the root zone. Also, the satellite product in their research is known to underestimate the timescales compared to in‐situ observations (Rondinelli et al., 2015; Shellito et al., 2016). When compared to storage values found for impervious surfaces by Wouters et al. (2015) (1.1–1.5 mm), the values in this study are higher as a result of the footprint scale analysis that includes natural in addition to impervious surfaces. Hence, the results show that both *λ* and *S*_0_ are at least five times smaller in all cities than in natural ecosystems indicating shorter timescales and lower storage capacities in urban areas regardless of their climate and vegetation fraction.

Since our method is based on direct inference from observations, the footprint of observation determines the area for which the storage is estimates and the reliability of the measurements is essential to the quality of our estimates. Since the fluxes are observed at neighborhood level, it is impossible to separate the (storage) source of ET. Further research could distinguish the different storage reservoirs by applying additional techniques like isotope analysis (Kuhlemann et al., 2021). The measurement reliability is insured by carefully selecting locations and applying quality control (Feigenwinter et al., 2012; Järvi et al., 2018; Velasco & Roth, 2010). All sites have an observation height well above the mean building height (see Table 1), and measure in the inertial sublayer. This reduces the variability in flux measurements in response to the heterogeneity of the monitored footprint, which is induced by the many, unevenly distributed surfaces with different characteristics and water storage capacities in the urban landscape. The only site in this research that includes a non‐homogeneous footprint is Seoul. The observations are filtered by wind direction to exclude a nearby forest. A relatively small variability between our estimates for each site suggest the observations are accurate enough for our application.

The methodology assumes that at the start of a drydown the storage capacity is completely full. A partly empty storage capacity would lead to an underestimation of the capacity, as less water is available for ET. We have compared the magnitude of the rain event before a drydown with the resulting parameters and found no correlation. Since the storage can be refilled by a series of events separated by dry days, we regressed the storage parameters against the Antecedent Precipitation Index (API; Fedora & Beschta, 1989). The API takes into account rainfall occurring during preceding days (here limited to 20), but its observed values show no correlations with the *λ* and *S*_0_. Therefore, the assumption of a completely filled storage is tangible and no selection has been performed based on rainfall event size. The evaporation directly after rainfall consists largely of interception ET from various surfaces (e.g., Gerrits, 2010; Grimmond & Oke, 1991; Oke et al., 2017). By calibrating an impervious‐storage parameterization (Wouters et al., 2015), estimated this storage to be between 1 and 1.5 mm for a site in Toulouse with little vegetation cover (8%), suggesting interception ET is an important component of urban ET also in more diverse and greener urban landscapes included in this study.

## 5 Conclusion

The timescales of ET recession observed through eddy covariance in urban environments appear to be considerably shorter than in rural environments. This is related to the storage capacity, which is also found to be lower. Based on 583 drydowns, we find recession timescales of cities within 1.8–20.1 days with the majority below 10.4 days and storage capacities between 1.3 and 28.4 mm with the majority below 13.4 mm. The timescales and storage capacities are inferred for the entire footprint (including all ET sources) and do not translate to impervious surfaces. All values found in urban areas are at least five times smaller than found in rural areas. We were unable to analyze differences between cities to vegetation fraction, local climate zone or climate for two reasons. First, the seasonal dependency in the storage capacities is as large as the total observed variation. Second, the number of sites is limited, and half of them contain data records shorter than 1 year. When provided with more data, the presented water storage capacity method has the potential to establish robust empirical relations explaining the differences between cities, in particular when complemented with soil moisture observations and/or Earth observation.

## Supporting information

Supporting Information S1

## Data Availability

The data that support the findings of this study are openly available in data.4tu at (http://doi.org/10.4121/13686973).
