# Supplementary material for: Urban Water Storage Capacity Inferred From Observed Evapotranspiration Recession
Source: Geophys Res Lett. 2022 Feb 8;49(3):e2021GL096069. doi: 10.1029/2021GL096069 (PMC9285425; doi:10.1029/2021GL096069)
Supplement: Supplementary file 1 — Supporting Information S1 [file GRL-49-0-s001.pdf]

Supporting Information for "Urban water storage capacity inferred from observed evapotranspiration recession"

H.J. Jongen[1,2], G-J. Steeneveld[2], J. Beringer[3], A. Christen[4], N. Chrysoulakis[5], K. Fortuniak[6], J. Hong[7], J-W. Hong[8], C.M.J. Jacobs[9,10], L. Järvi[11,12], F. Meier[13], W. Pawlak[6], M. Roth[14], N.E. Theeuwes[5,16], E. Velasco[17], R. Vogt[18]and A.J. Teuling[1]

[1]Hydrology and Quantitative Water Management, Wageningen University, Wageningen, The Netherlands.

[2]Meteorology and Air Quality, Wageningen University, Wageningen, The Netherlands.

[3]School of Agriculture and Environment, University of Western Australia, Crawley, Australia.

[4]Chair of Environmental Meteorology, Faculty of Environment and Natural Resources, University of Freiburg, Freiburg, Germany

[5]Foundation for Research and Technology Hellas, Institute of Applied and Computational Mathematics, The Remote Sensing Lab, Heraklion, Crete, Greece

[6]Department of Meteorology and Climatology, Faculty of Geographical Sciences, University of Łódź, Łódź, Poland.

[7]Department of Atmospheric Sciences, Yonsei University, Seoul, South Korea.

[8]Korea Environment Institute, Sejong, South Korea.

[9]Wageningen Environmental Research, Wageningen University and Research, Wageningen, The Netherlands.

[10]National Institute for Public Health and the Environment (RIVM), Bilthoven, The Netherlands

[11]Institute for Atmospheric and Earth System Research / Physics, University of Helsinki, Helsinki, Finland.

[12]Helsinki Institute of Sustainability Science, University of Helsinki, Hesinki, Finland.

[13]Chair of Climatology, Technische Universität Berlin, Berlin, Germany.

[14]Department of Geography, National University of Singapore, Singapore.

January 10, 2022, 9:20am

[15]Department of Meteorology, University of Reading, Reading, United Kingdom.

[16]Royal Netherlands Meteorological Institute (KNMI), De Bilt, The Netherlands.

[17]Independent Research Scientist, Singapore.

[18]Atmospheric Sciences, Department of Environmental Sciences, University of Basel, Basel, Switzerland

Contents of this file

1. Figures S1 to S5
2. Table S1

Introduction This supplementary information contains additional figures and one table further visualizing the analyses that we present in the paper. We include the results of the urban water storage capacity estimation approach with a correction for the amount of available solar energy (Figure S1 and S2 and Table S1). We also present the comparison of the site characteristics with the estimated parameters related to the water storage capacity (Figures S3 and S4), and a more detailed comparison of the vegetation fraction with the estimated parameters (Figure S5).

Corresponding author: H.J. Jongen, Hydrology and Quantitative Water Management Group, Wageningen University, Droevendaalsesteeg 3, Wageningen, 6708PB, The Netherlands. (harro.jongen@wur.nl)

Table S1. Same as last part of Table 1, but with results from the analysis with ET corrected for the amount of available solar energy.

City	Drydowns	Days	ET_0 (mm d^{-1})	λ (day)	$t_{\frac{1}{2}}$ (day)	S_0 (mm)	R^2
Amsterdam	16	61	0.6 – 2.1 (1.5)	2.8 – 7.6 (5.2)	1.9 – 5.2 (3.6)	3.9 – 14.8 (6.6)	0.60
Arnhem	39	148	0.9 – 1.3 (1.1)	1.6 – 2.9 (2.2)	1.1 – 2.0 (1.6)	2.1 – 3.2 (2.6)	0.80
Basel (AESC)	109	445	0.9 – 1.2 (1.1)	4.1 – 5.2 (4.7)	2.8 – 3.6 (3.3)	3.9 – 5.4 (4.7)	0.75
Basel (KLIN)	150	623	1.2 – 1.4 (1.3)	5.5 – 7.2 (6.3)	3.8 – 5.0 (4.4)	6.4 – 9.3 (7.4)	0.66
Berlin (ROTH)	9	36	0.6 – 1.9 (0.8)	4.8 – 13.7 (11.9)	3.3 – 9.5 (8.2)	4.2 – 22.1 (11.5)	0.79
Berlin (TUCC)	30	122	0.4 – 0.9 (0.6)	2.4 – 4.0 (2.8)	1.7 – 2.8 (2.0)	1.2 – 3.1 (1.8)	0.68
Helsinki	41	177	1.7 – 2.0 (1.8)	3.4 – 7.8 (5.0)	2.4 – 5.0 (3.5)	6.6 – 11.9 (8.6)	0.80
Heraklion (HECKOR)	3	13	0.9 – 3.4 (2.9)	0.8 – 5.0 (1.7)	0.6 – 3.5 (1.2)	1.5 – 14.3 (2.9)	0.86
Lodz	55	249	1.3 – 1.8 (1.6)	3.2 – 4.8 (3.9)	2.2 – 3.3 (2.7)	4.2 – 7.6 (5.5)	0.70
Melbourne	2	9	0.7 – 1.8 (1.2)	1.6 – 10.2 (5.9)	1.1 – 7.1 (4.1)	1.1 – 17.9 (9.5)	0.65
Mexico City	9	52	0.8 – 1.5 (1.4)	4.8 – 14.6 (9.5)	3.3 – 10.1 (6.6)	5.6 – 19.1 (11.4)	0.60
Seoul	7	39	1.1 – 2.7 (1.7)	1.7 – 8.2 (4.3)	1.2 – 5.7 (3.0)	5.5 – 9.7 (8.9)	0.53
Singapore	8	43	1.3 – 1.6 (1.4)	6.2 – 17.7 (8.8)	4.3 – 12.3 (6.1)	9.3 – 24.6 (12.5)	0.76
Vancouver	61	282	1.3 – 1.7 (1.4)	4.9 – 7.8 (6.1)	3.4 – 5.4 (4.2)	6.7 – 10.0 (7.7)	0.60

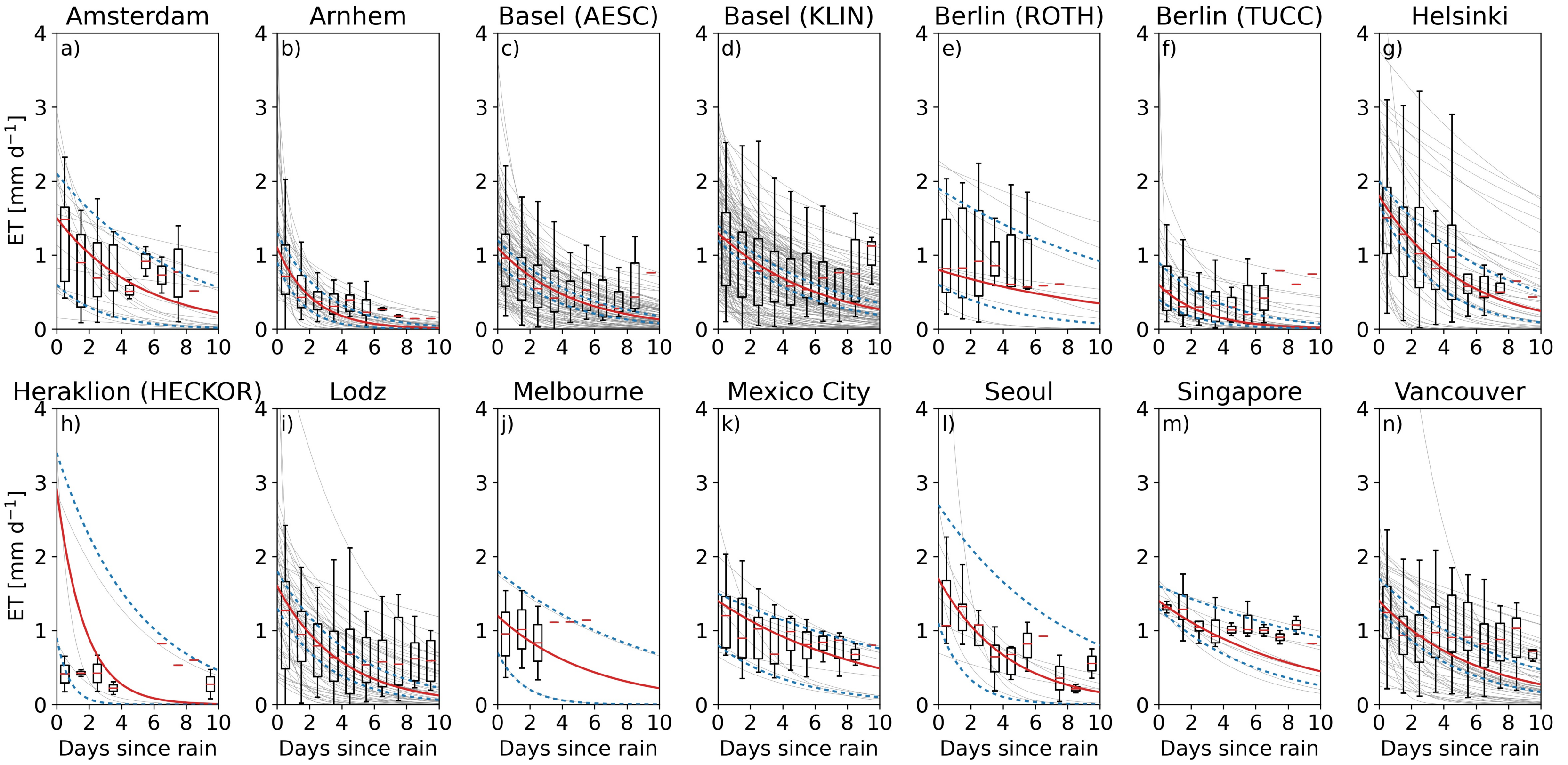

Figure S1. Same as Figure 2, but with results from the analysis with ET corrected for the amount of available solar energy. This correction is performed by multiplying the evaporative fraction by the average available energy over the drydown.

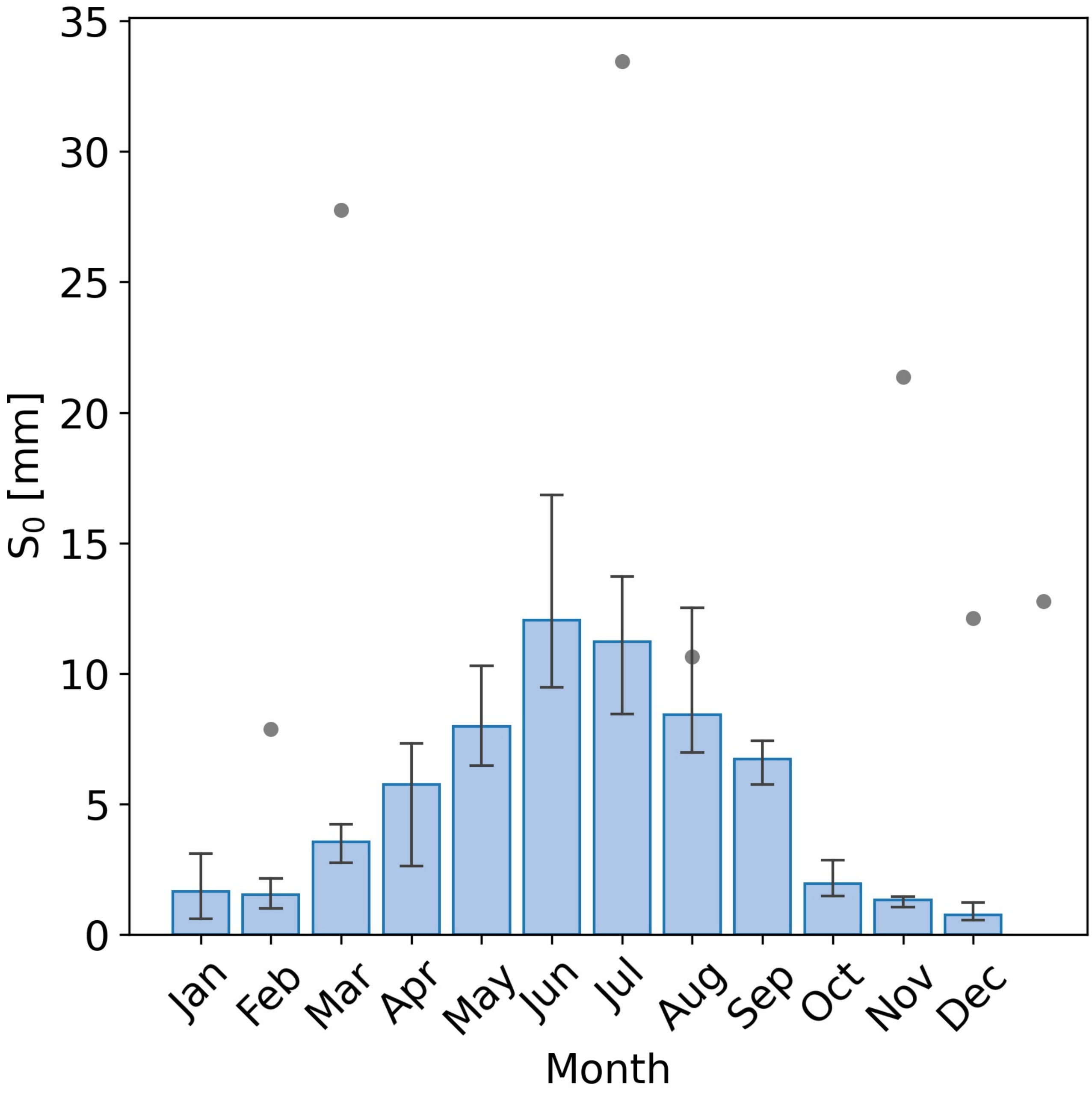

Figure S2. Same as Figure 3, but with results from the analysis with ET corrected for the amount of available solar energy. This correction is performed by multiplying the evaporative fraction by the average available energy over the drydown.

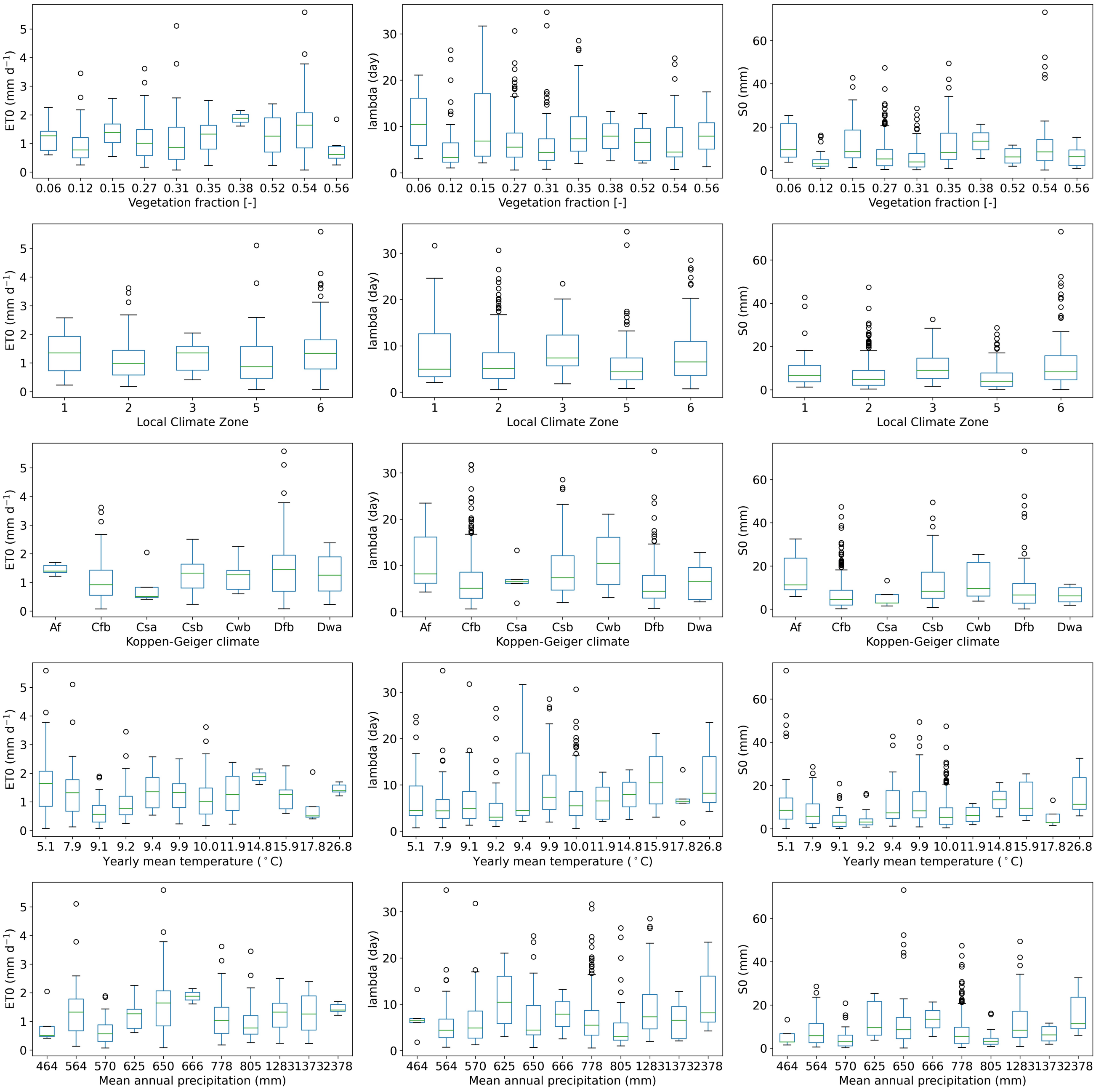

Figure S3. Estimated model parameters as function of climatological and urban form site characteristics.

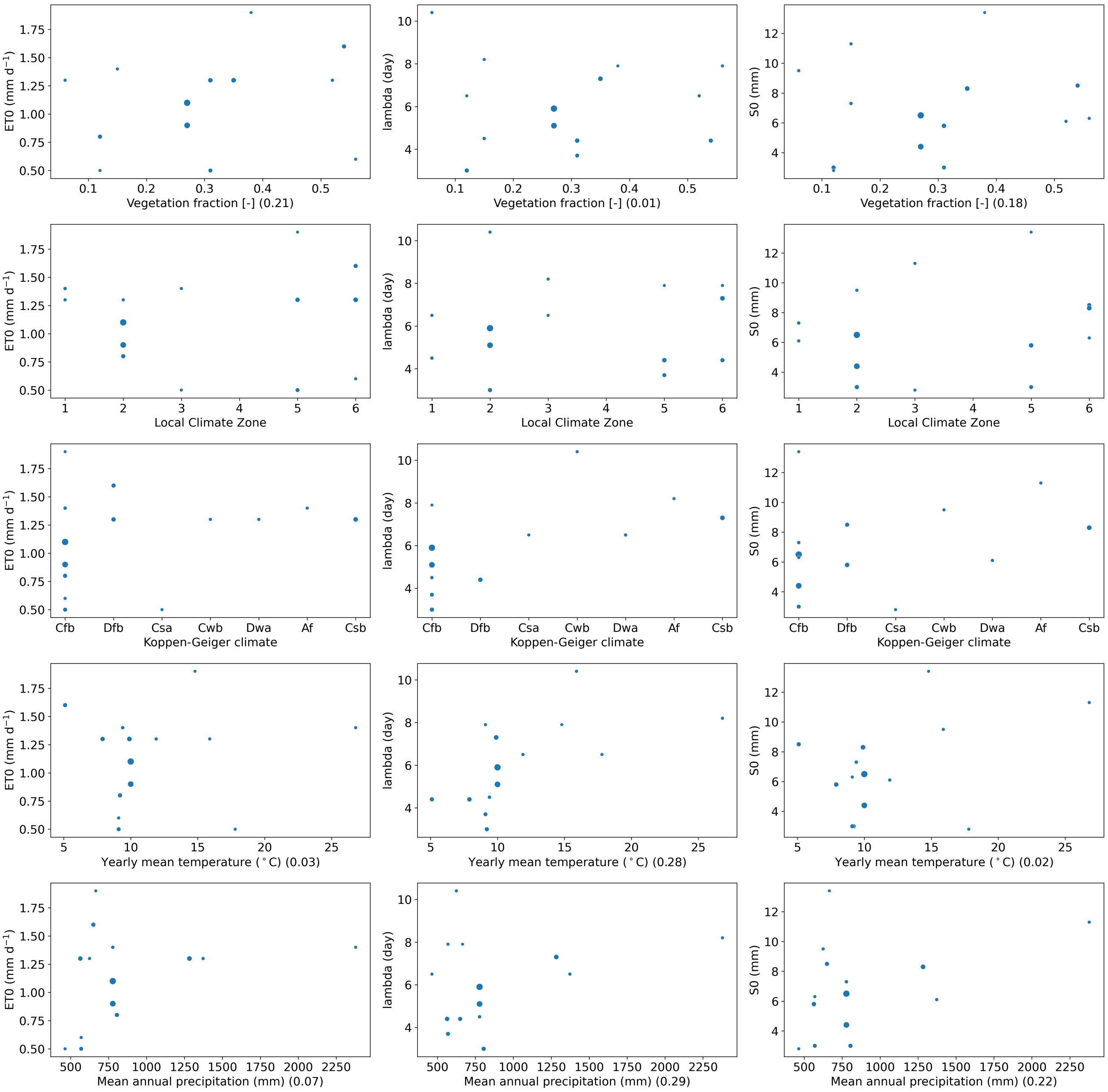

Figure S4. Estimated model parameters as function of climatological and urban form site characteristics. The size of the dots indicates the number of drydowns. Between brackets the correlation coefficient is displayed based on a weighted linear regression (based on the number of drydowns per city) for the quantitative site characteristics.

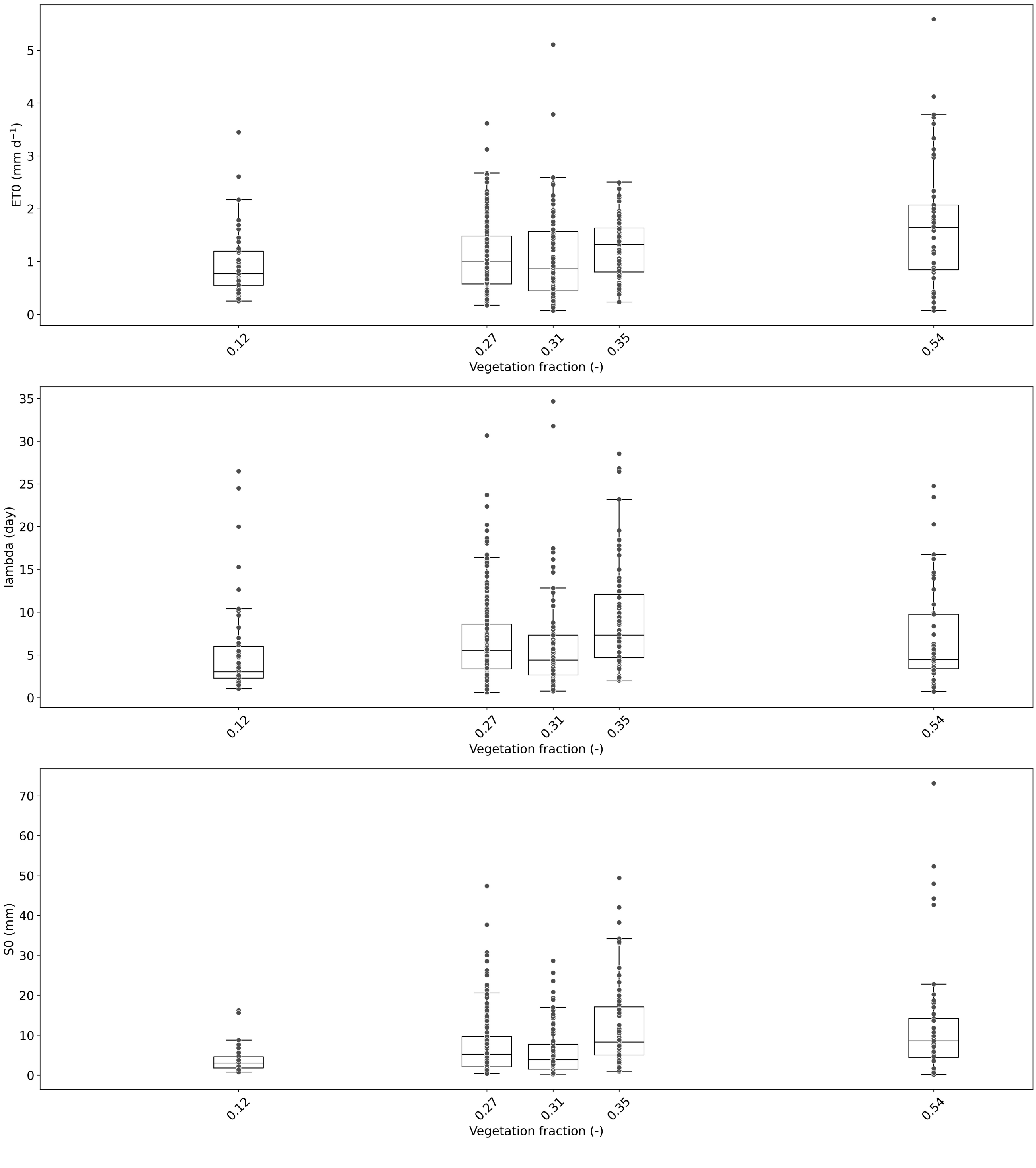

Figure S5. Boxplots of estimated model parameters as function of vegetation fraction. Only locations with at least 20 drydowns are taken into account.
